# Commentary: CiteSpace-based visualization of nutritional research hotspots for oncology patients

**DOI:** 10.3389/fnut.2026.1838665

**Published:** 2026-05-21

**Authors:** Paolo Pedrazzoli, Elisa Mattavelli, Lorenzo Perrone, Alberto Ambrosio, Riccardo Caccialanza

**Affiliations:** 1 Department of Internal Medicine and Medical Therapy, University of Pavia, Pavia, Italy; 2 Department of Oncology – Comprehensive Cancer Center, Fondazione IRCCS Policlinico San Matteo, Pavia, Italy; 3 Clinical Nutrition and Dietetics Unit, Department of Oncology – Comprehensive Cancer Center, Fondazione IRCCS Policlinico San Matteo, Pavia, Italy; 4 Department of Health Management, Fondazione IRCCS Policlinico San Matteo, Pavia, Italy; 5 Department of Oncology and Hemato-Oncology, University of Milan, Milan, Italy

**Keywords:** bibliometric, cancer, nutritional assessment, nutritional support, visual analysis

We read with great interest the recent bibliometric analysis on nutritional research in oncology patients by Zhang et al. (1). The authors provide a comprehensive overview of global trends, highlighting the increasing relevance of nutritional assessment, sarcopenia, and peri-treatment interventions.

The main limitations of this study, including the decision to retrieve records exclusively from Web of Science and the specific CiteSpace parameter settings, were appropriately acknowledged, thereby enhancing the transparency and interpretability of the findings. We aim to highlight two key methodological and interpretative limitations that, in our view, merit further discussion: first, the potential underrepresentation of nutritional support interventions due to the adopted search strategy; and second, the interpretation of geographical and institutional productivity, particularly regarding the limited visibility of Europe.

The first issue concerns the scope of the search strategy. This study's English-language literature search strategy frames “nutritional research” largely through assessment-related terminology, including terms such as “nutrition OR nutritional status,” “nutritional assessment,” and “malnutrition.” While this approach provides a clear and valuable representation of the nutritional assessment domain, it may not fully capture another essential component of nutritional research in oncology: nutritional support interventions. This includes areas such as medical nutrition therapy and targeted nutritional strategies (e.g., nutritional counseling and oral nutritional supplements) aimed at improving clinical outcomes (2).

This limitation is relevant, as the selected keywords in bibliometric analyses shape network mapping, thus influencing the way a research field is interpreted. Without specific intervention-related terms in the search strategy, studies focused on nutritional interventions are likely to be excluded. As a result, the broad field of nutritional research in oncology may be represented predominantly through the lens of nutritional assessment, leading to an underestimation of nutritional intervention research.

In addition to the implications of the search strategy, a second concern relates to the interpretation of geographical and institutional productivity. The analysis focuses primarily on institutional scientific productivity. While it is accurate to report that the most productive institutions are predominantly located in China, North America, and Australia, this should not be considered a straightforward proxy for region-level scientific contribution. While only marginally addressed in the original study, this issue is particularly relevant for Europe, which has historically been one of the leading regions in clinical nutrition in oncology. Within this tradition, equal importance is placed on both nutritional assessment and the implementation of tailored nutritional support strategies (3–5). Supporting this perspective, a bibliometric analysis published in 2023 focusing specifically on medical nutrition therapy in oncology demonstrated a substantial European publication output, including notable contributions from France, the United Kingdom, Germany, and Italy (6). However, when such analyses are translated to the institutional level, the results appear more consistent with those reported by Zhang et al. (1), where only one European institution appears among the top contributors: Fondazione IRCCS Policlinico San Matteo, Pavia, Italy (7).

Taken together, these considerations have implications for future bibliometric analyses in oncology nutrition, which should adopt search strategies able to capture both assessment- and intervention-oriented research while also interpreting geographical productivity across complementary institutional, national, and regional levels.

It is important to emphasize that the presence of a single European institution among the top contributors should not be interpreted as a matter of prestige or isolated institutional excellence, but rather as a signal of a broader structural issue. As also mentioned by the authors, European research activity in this field appears fragmented, often organized in smaller consortia with limited visibility in global bibliometric analyses. In contrast to the more centralized funding and coordinated research networks seen in China and the United States, Europe may lack sufficiently integrated, large-scale collaborative frameworks capable of generating high-impact, high-volume outputs in oncology nutrition (8).

Given the growing importance of nutritional management in cancer care (2), particularly in areas such as sarcopenia, prehabilitation, and outcome-oriented interventions, this limited visibility raises important concerns. It suggests that Europe may be at risk of playing a secondary role in shaping future standards of care, despite its strong clinical tradition and leadership in clinical guidelines (3–5).

These findings highlight the urgent need for greater coordination, visibility, and integration of European research efforts. Strengthening European collaboration through multicenter prospective trials, shared registries, and coordinated funding initiatives could significantly enhance both scientific impact and clinical translation.

In this context, the experience of Fondazione IRCCS Policlinico San Matteo, which is listed among high-performing European institutions (1), may provide contextual insight and highlight the need for more coordinated efforts at the European level.
